# Event-Based Circular Detection for AUV Docking Based on Spiking Neural Network

**DOI:** 10.3389/fnbot.2021.815144

**Published:** 2022-01-12

**Authors:** Feihu Zhang, Yaohui Zhong, Liyuan Chen, Zhiliang Wang

**Affiliations:** School of Marine Science and Technology, Northwestern Polytechnical University, Xi'an, China

**Keywords:** DVS, SNN, AUV, Hough transform, P4P, docking

## Abstract

In this paper, a circular objects detection method for Autonomous Underwater Vehicle (AUV) docking is proposed, based on the Dynamic Vision Sensor (DVS) and the Spiking Neural Network (SNN) framework. In contrast to the related work, the proposed method not only avoids motion blur caused by frame-based recognition during docking procedure but also reduces data redundancy with limited on-chip resources. First, four coplanar and rectangular constrained circular light sources are constructed as the docking landmark. By combining asynchronous Hough circle transform with the SNN model, the coordinates of landmarks in the image are detected. Second, a Perspective-4-Point (P4P) algorithm is utilized to calculate the relative pose between AUV and the landmark. In addition, a spatiotemporal filter is also used to eliminate noises generated by the background. Finally, experimental results are demonstrated from both software simulation and experimental pool, respectively, to verify the proposed method. It is concluded that the proposed method achieves better performance in accuracy and efficiency in underwater docking scenarios.

## 1. Introduction

**Figure 1:**
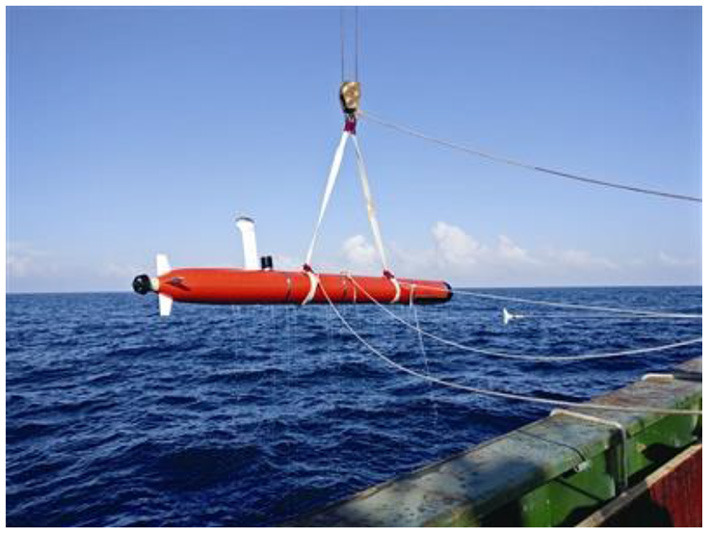
Autonomous underwater vehicle.

Although the exploitation of ocean resources has attracted significant interests from both industrial and societal, the development of marine science and technology still suffers limited activities (Saeki, 1985). Exploring underwater environments presents many problems, such as water pressure changing and oxygen supplying (Stachiw, 2004). Autonomous Underwater Vehicles (AUV) (Figure 1), often referred to the Unmanned Underwater Vehicles (UUV), have been developed along with the rapid exploitation of the ocean, and leading to a reduction in operational costs.

However, due to the volume and mass issues, AUV carries limited energy (Chiche et al., 2018). It is challenging to perform better in large-scale environments, and the AUV is often required to replenish energy and transmit information frequently. Therefore, the underwater docking technique is developed to provide powerful energy supply, information processing and communication support for AUVs (Benton et al., 2004). To the best knowledge of authors, almost all underwater docking tasks rely on optical cameras for short-range pose estimation between AUV and docking station (Wang et al., 2016). The docking system developed by Woods Hole Oceanographic Institute for Remus series AUV (Stokey et al., 2001) and the docking system designed by MBARI for bluefine AUV (McEwen et al., 2008) are two typical inclusive docking systems. In docking process, the guidance system plays a vital role concerning the whole system, while visual perception contributes a lot in short-range docking (Zhao et al., 2013).

In Zhong et al. (2019), a binocular localization method for AUV docking is presented, and an adaptively weighted OTSU method is developed for feature extraction, the operation frequency of which is about 10 Hz. Jointly, in Wang et al. (2016), gray-scale feature analysis, edge detection and morphology methods are used to improve the algorithm of calculating the centers of target lights. Furthermore, in Yan et al. (2019), a visual positioning algorithm based on the L-shaped light array is proposed. Previous studies generally used the frame-based camera to carry out detection, which contains redundant background data, lacks solutions to high exposure as well as high-speed response capability such as more than 1kHz.

As frame-based camera also suffers the over-exposure issue while close to the docking light, it is quite challenging to determine the surrounding environment and its own pose. Considering the instability of underwater motion, it is also difficult to keep relatively stationary. As a result, motion blur could not be directly eliminated. In contrast to frame-based cameras, the event-based camera is sensitive to dynamic information and suitable for moving target recognition. In Piatkowska et al. (2012), an algorithm for spatiotemporal tracking to detect moving persons that is suitable for DVS was proposed. In Chen (2018), discriminative knowledge was transferred from a frame-based convolutional neural network (CNN) to the event-based modality via intermediate pseudo-labels, and then supervised learning was combined to detect cars. In Seifozzakerini et al. (2016), a method using Hough transform and event-based clustering algorithm to track multiple lines was proposed. So far, most research in underwater applications still relies on frame-based cameras, whereas the event-based visual perception method has not been well-explored.

In this paper, an event-based detection of multiple circles for AUV Docking based on the spiking neural network method is proposed. The main contributions of this work are concluded as follows:

First, the proposed approach significantly eliminates the redundant information during the docking task. The frame-based camera produces information on the whole image. However, in underwater scenarios, most of the image backgrounds are adaptively filtered by the event-based camera. Thus, the computation performance is guaranteed with respect to the on-chip resource in the AUV.
Second, based on the Spiking Neural Network, the relative position information is acquired between the AUV and the docking ring. Furthermore, the PnP algorithm and a spatiotemporal filter are simultaneously utilized to estimate the relative depth and reduce the noise interference caused by vibration and background activities, respectively.
Third, the proposed approach keeps robust in complex underwater environments. The event-based camera could effectively eliminate motion blur and over-exposure, which is a natural advantage in underwater docking applications.
The structure of this paper is organized as follows: section 2 briefly introduces the backgrounds. Section 3 investigates the preprocessing work with respect to spatiotemporal filter. Section 4 presents the SNN detection framework and section 5 exhibits experimental results. Finally, this paper is concluded in section 6.

## 2. Background

**Figure 2:**
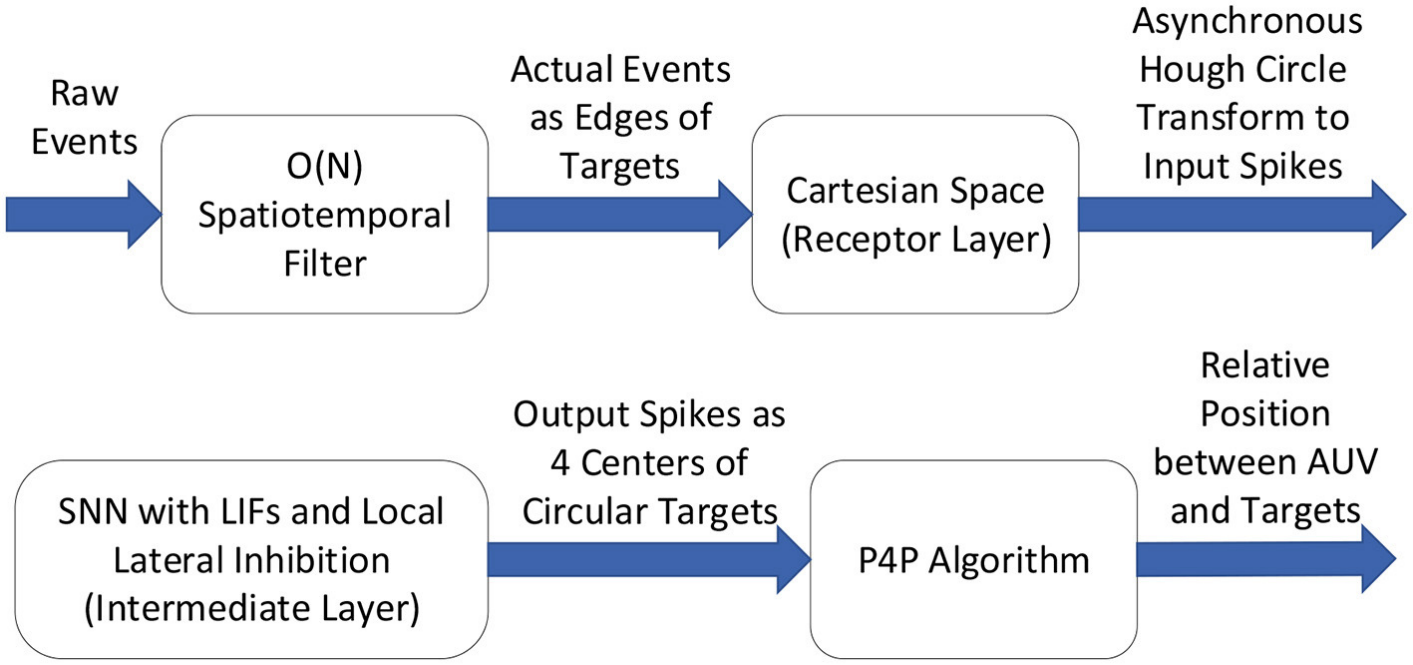
Overview of the detection method.

In this paper, the overview of the proposed detection method could be briefly expressed in Figure 2, which is based on asynchronous Hough circle transform and the theory of Spiking Neural Network.

### 2.1. Dynamic Vision Sensor

The Dynamic Vision Sensor (DVS, also called event-based camera) is a neuromorphic camera that behaves similar to the human visual system by modeling the human retina (Lichtsteiner Patrick and Tobi, 2008). In contrast to the frame-based camera, which captures and transmits frames synchronously at a fixed frame rate, DVS keeps super performances by asynchronously transmitting events as soon as each occurs in a pixel. Once the logarithmic intensity change of a pixel is larger than a predefined threshold, an event of the corresponding polarity will asynchronously generate depending on the direction of the change of brightness (Seifozzakerini et al., 2016). Hence, it is sensitive to intensity logarithmic change. However, the information of the magnitude change is not transmitted. Every event consists of four parameters (*t, x, y, p*) including the timestamp *t* in μ*s*, position (*x, y*) in pixels and polarity *p* which is binary (+/−), where parameters *t*,*x*, and *y* are integer values. Each pixel sensor is independent of the other pixel sensors so that its own intensity change can be adapted, and a very high dynamic range of DVS is formed. DVS outputs compressed digital data as a tuple, avoiding redundancy and latency caused by conventional cameras.

### 2.2. Spiking Neuron Model and Spiking Neural Network

Spiking neural network (SNN) is the third generation of neural network models, improving the realistic level of neural simulation (Maass, 1997). Each Spiking Neuron receives some spike inputs and generates one spike output (Burkitt, 2006). The input is a sequence of spikes that happen at different times, increasing or decreasing the neurons' Membrane Potential (MP). Moreover, MP is constantly decaying linearly till zero. Whenever the MP exceeds the positive or negative threshold (only a positive threshold considered to simplify the calculation), a spike is produced as output. Later MP of the neuron and its local lateral neighbors are inhibited to zero and then enter a short no response period.

In this paper, the leaky integrate-and-fire (LIF) (Brunel and Sergi, 1998) spiking neurons are considered to establish SNN. An SNN representing the parameter space is also utilized to detect circle objects based on the asynchronous Hough transform. The neurons' inputs are the pulses generated by Hough transform mappings, and the output neurons' coordinates are the pixel coordinates of the object centers. Algorithm 1 shows this neuron model, where *t*_*i*_ is the current timestamp, *t*_*i*−1_ is the last time the neuron is stimulated, *t*_*d*_ is the attenuation duration, *u*_*i*_ is the MP at time *t*_*i*_, *sign*(*u*_*i*−1_) is the positive or negative signal of the potential at the previous time and *s*_*i*_ is the input spike at time *t*_*i*_.

**Algorithm 1:** Updating procedure of a spiking neuron when receiving an input spike.

Initialize the spike value *s*_*i*_ = 1*mv*, rate of decay		
λ = 0.0006*mv*/μ*s*, and spike threshold *u*_*th*_ = 150*mv*		
**for** every input spike *s*_*i*_ at time *t*_*i*_ **do**		
	*t*_*d*_ = *t*_*i*_ − *t*_*i* − 1_	
	*u*_*i*_ ← *sign*(*u*_*i*−1_) · *max*(|*u*_*i*−1_| − λ · *t*_*d*_, 0)	
	*u*_*i*_ ← *u*_*i*_ + *s*_*i*_	
	**if** |*u*_*i*_| ≥ *u*_*th*_ **then**	
		Generate output spike δ = *sign*(*u*_*i*_) at *t*_*i*_
		Inhibit all connected neurons in local area
		*u*_*i*_ ← 0
	Update *t*_*i*_ to new timestamp	

## 3. Spatiotemporal Filter for Reducing Noise

In fact, small changes in the lower intensity of a pixel often lead to an apparent change which may generate an event afterward. Hence, in darker places, more and more noisy events will be produced (Seifozzakerini et al., 2016). However, the frame-based filtering algorithm is not suitable for DVS. As Hough Transform (Illingworth and Kittler, 1987) is sensitive to noisy measurements, a spatiotemporal filter is utilized to process raw events before the detection phase.

Background Activity (BA) noise is expected in the event stream, which is produced by thermal noise and junction leakage currents (Lichtsteiner Patrick and Tobi, 2008). However, unlike actual events, BA events lack time correlation with other events in their spatial neighborhood. Besides, the BA events are proved to correspond to Poisson distribution (Khodamoradi and Kastner, 2021), the probability of a known number *n* of events if all those events are independent and happen at a given average rate λ:


(1) $$\begin{array}{l} P\left\{n\right\}=\frac{\lambda^{n}\cdot e^{-\lambda }}{n!} \end{array}$$

In order to recover interested events from raw data, the spatiotemporal filter also records the early timestamps. Once an event is processed, the filter searches the corresponding spatial neighborhood. If the timestamp difference between two adjacent events in the very near spatial coordinate is found less than a threshold *dt*, it is regarded as an actual event. Otherwise, it is discarded. The principle could be briefly expressed as follows:


(2) $$\begin{array}{l} e(t_{i},x_{i},y_{i},s_{i})\notin BA\;noise\Leftrightarrow \\ \;\exists |t-t_{mn}|\leq dt,\\ s.t.|m-x|\leq 1\cap |n-y|\leq 1 \end{array}$$

Where *e* is the new event to be processed, *t*_*mn*_ is the timestamp of the last event at the position (*m, n*) not including the new event, *dt* is the time threshold.

The Spatiotemporal filter proposed by Alireza Khodamoradi (Khodamoradi and Kastner, 2021) is suitable for embedded applications and moving cameras, which is applied in this paper. It uses only two memory cells for recursively filtering the image, which can significantly eliminate memory requirements. The computational performance is thus reduced from *O*(*N*^2^) to *O*(*N*). Meanwhile, this filter increases the data density of real events by 180%.

## 4. Event-Based Multiple Circle Detection and Pose Estimation

As mentioned above, optical guidance plays an essential role in close range, and the AUV is usually guided by lights mounted around the docking station. It is observed that using single lights makes the task challenging, as the 3D pose information is always missing. Therefore, at least three light sources are required for AUV docking. In this paper, four circle shape LED lights are utilized to overcome the aforementioned issue. From an underwater perspective, each circular light looks like a dot from long distances and a circle from nearby places. Furthermore, the halo often appears sparse compared to the dense light source, and the corresponding position includes bias regarding the light sources. In order to improve the localization accuracy, the position of the light source should be considered instead of the halo. Hence, the spatiotemporal noise filter is utilized to eliminate the halo around.

In this paper, the detection of multiple lights is performed using Hough transform (Hough, 1959). Note that the sparse events data has already been acquired with redundancy, while the temporal asynchrony process is implemented afterward. According to the difference between frame-based cameras and DVS that gradient information is challenging to obtain from frame-free events, traditional Hough circle transform is adapted to proceed asynchronous events. A simple way to solve this problem is to accumulate all events in a period into a pseudo frame, and then the conventional frame-based gradient Hough transform (Chen et al., 2012) can be used. However, ignoring the time information of every event like that will reduce the sensitivity and dynamic characteristics of the algorithm. To achieve this goal, an asynchronous Hough circle transform based on Spiking Neural Network is thus proposed to accurately and effectively detect underwater lights.

### 4.1. The Proposed SNN for Asynchronous Hough Circle Transform

Pixels are simultaneously generated on frame-based cameras. However, the stream from the event-based camera is asynchronous; that is, events are generated with time sequence. In order to take advantage of event streams, every single event must be processed asynchronously. In this paper, new events are processed immediately without being accumulated as a frame. Therefore, the asynchronous Hough transform algorithm in SNN is proposed to effectively identify the sparse events in the time scale of microseconds.

In this paper, we propose the SNN model with Hough Transform to detect multiple circle objects. As shown in Seifozzakerini et al. (2016), a straight line is detected based on a 2-dimensional SNN model, which only contains two parameters: distance ρ and angle θ. However, for circular feature detection, three parameters (*x*-coordinates, *y*-coordinates, and radius *r*) should be taken into account. Once the radii are unknown, the event-based Hough transform should be performed in 3D space (*x*_*c*_, *y*_*c*_, *r*). Ni et al. (2012) extracted the microspheres with known radius, but without utilizing the characteristics of SNN. To extend both the number and radius of potential objects, Hough circle transform and SNN are jointly utilized. Noting that constructing a 3D SNN leads to huge computation resources, only two parameters *x*_*c*_ and *y*_*c*_ are selected to avoid the computational cost. Here, the range of parameter *r* is manually selected outside the SNN. The process is as follows:

**First**, conduct a spatiotemporal filter (in section 3) with raw events to eliminate the noises and halos around the underwater lights.
**Second**, the Hough circle transform algorithm based on asynchronous events is utilized to map events from Cartesian coordinate space to 2D parameter space.

By continuously fetching the latest event *e*_*i*_ = (*t*_*i*_, *x*_*i*_, *y*_*i*_, *s*_*i*_) from the flow queue, *P*_*t*_*n*__ is defined as a collection of points generated at timestamp *t*_*n*_:


(3) $$\begin{array}{l} P_{t_{n}}=\left\{\left(y_{i},x_{i}\right)|\exists e_{i}\left(t_{i},x_{i},y_{i},s_{i}\right)\right\} \end{array}$$

For each acquired event, the coordinates (*y*_*i*_, *x*_*i*_) are extracted, and the mapping from the Cartesian coordinate space to the Hough parameter space is performed. Note that the Hough transformation for each event is asynchronously processed. Especially every time an event produces mappings to the circle centered on itself with radius *r* and central angle θ_*i*_ from 0 to 360 degrees. The radius range is set from *r*_*min*_ to *r*_*max*_ aiming to detect different sizes. Therefore, 360 · (*r*_*max*_ − *r*_*min*_) mappings are generated for one event at its timestamp *t*_*i*_. Mappings from every event which occurs at a circle's edge would include one mapping at its center (*y*_*c*_, *x*_*c*_). Thus the calculation formulas of the Hough circle transform are calculated as follows:


(4) $$\begin{array}{l} \begin{array}{l} \;x_{c}=x_{i}+r\cdot cos\theta_{i}\\ \;y_{c}=y_{i}+r\cdot sin\theta_{i}\\ s.t.\;r\in \left[r_{min},r_{max}\right],\theta_{i}\in \left[0,360\right) \end{array} \end{array}$$

Where *x*_*c*_ and *y*_*c*_ are the horizontal and vertical coordinates of the center, respectively. *r* is radius,θ_*i*_ is the central angle from (*y*_*i*_, *x*_*i*_) to (*y*_*c*_, *x*_*c*_), and (*y*_*i*_, *x*_*i*_) ∈ *P*_*t*_*n*__. Mappings outside the range scope are not considered.

**Third**, improve Hough transformation with SNN for circular detection.

It is observed from event streams that neurons at the object location output spikes. Hence laterally suppressing adjacent neurons make the detection task possible. Considering that the original Hough parameter space does not contain time information, a time-sensitive SNN is constructed as an intermediate layer for asynchronous processing. The input spikes of SNN are all mappings generated by asynchronous Hough transform, and the output spike is the pixel coordinates of targets.

A M × N SNN is established by using the LIF spiking neuron model (section 2.2), where M and N represent the parameter range of Y-coordinate and X-coordinate of the circle's center, respectively. The height and width of SNN can be selected according to the resolution of the camera. The membrane potentials of all neurons are initialized as 0 at the beginning. Meanwhile, a matrix is utilized to update the timestamp *t*_*i*_ of each event, which is initialized by the first incoming event's timestamp. Noticed that each event in the receptor layer inputs spikes *s*_*i*_ to 360 · (*r*_*max*_ − *r*_*min*_) neurons (*y*_*c*_, *x*_*c*_) in the intermediate layer, and each spike input increases the absolute value of MP regards to the neuron, whereas the MP always decreases with a fixed linear rate λ. The residual MP is calculated by MP at the last time *t*_*i*−1_ minus the decay value and adds the current spike value. *t*_*d*_ is the time duration between the current spike input time *t*_*i*_ and the last spike input time *t*_*i*−1_. MP will not attenuate after decay to 0. The events can therefore be mapped to SNN by using Equation (4) and defined as follow:


(5) $$\begin{array}{l} Hough\left(P_{t_{n}}\right):\;P_{t_{n}}\to SNN_{t_{n}} \end{array}$$

where *SNN*_*t*_*n*__ is a matrix that changes over time:


(6) $$\begin{array}{l} SNN_{t_{n}}(y_{c},x_{c})=\{\begin{array}{l} SNN_{t_{n-1}}(y_{c},x_{c})+|s_{i}|\;if\;\exists Hough(P_{t_{n}})\\ SNN_{t_{n-1}}(y_{c},x_{c})-\lambda \cdot t_{d}\;whenever\\ \;SNN_{t_{n-1}}(y_{c},x_{c})>0 \end{array}\\ \;for\;\forall y_{c}\in [0,rows),\forall x_{c}\in [0,columns),s_{i}=\pm 1. \end{array}$$

The intermediate layer mapped by all incoming events can be expressed as *Hough*(*P*_*t*_*n* − *k*+1_, *t*_*n*+1__), where *t*_*n*+1_ is the current time, *s*_*i*_ is the input spike, λ is the rate of decay, and *t*_*d*_ is decay time. The following recursive formula for continuous conversion with time is utilized, which is also called continuous SNN based Hough mapping:


(7) $$\begin{array}{l} \begin{array}{l} Hough\left(P_{t_{n-k+1},t_{n+1}}\right)=Hough\left(P_{t_{n-k},t_{n}}\right)\\ \;+Hough\left(P_{t_{n+1}}\right)-Hough\left(P_{t_{n-k}}\right)\\ \;Hough\left(P_{t_{n-k},t_{n}}\right)=\overset{t_{n}}{\underset{i=t_{n-k}}{\sum }}Hough\left(P_{t_{i}}\right) \end{array} \end{array}$$

The continuous Hough mapping is performed in the intermediate layer of SNN. Once the value of MP exceeds the positive threshold, the neuron is activated and outputs a positive pulse at (*x*_*c*_, *y*_*c*_) at timestamp *t*_*i*_ to the output layer. Then MP is reset to zero. Afterward, the neuron enters a short period of no response, during which the input pulse is ignored to avoid multiple outputs originating from the same target. Meanwhile, the activated neurons inhibit the MP of other neurons within the local lateral margin of *m* × *m* to reduce duplicate detections of one object. The MP of non-activated neurons and neurons not near activated neurons are not inhibited and still decay naturally. Once a neuron receives a pulse, it updates the timestamp of the corresponding coordinates within the timestamp matrix. The updating procedure of a neuron when receiving an input spike is shown in Algorithm 1 (section 2.2).

As the original polarity *s*_*i*_ of the event is divided into positive and negative, the two symmetrical edges of the target generate spiking inputs with opposite polarities, which leads to false alarms. Therefore, the absolute value of spikes is used in calculation, while all inputs are considered positive spikes.

**Figure 3:**
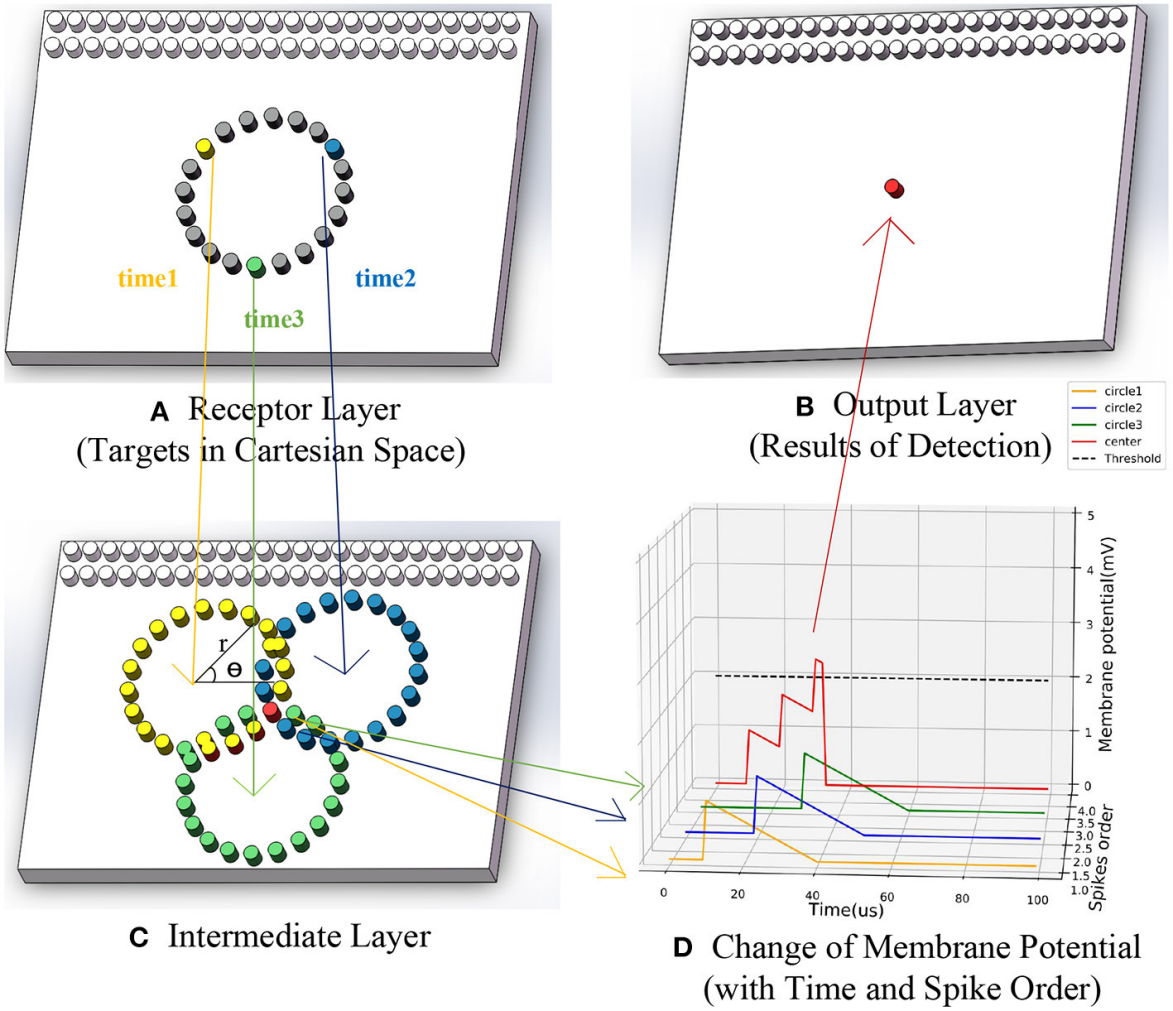
The running process of SNN based asynchronous hough circle transform. **(A)** Receptor layer (target in Cartesian space). **(B)** Output layer (results of detection). **(C)** Intermediate layer. **(D)** Change of membrane potential (with time and spike order).

Once a neuron (*y*_*c*_, *x*_*c*_) outputs a spike to the output layer at *t*_*i*_, the position of the center is detected. All spikes output in a period *t*_*n*−*k*_*t*_*n*_ are counted, that is, all targets detected during that time. The process of SNN could be briefly shown in Figure 3. Firstly, the receptor layer's yellow, blue, and green events generated input spikes with time sequence. Secondly, the MP of corresponding neurons in the intermediate layer changed in turn. Finally, a red spike outputs afterward.

**Fourth**, matching feature to the rectangular target with the following rules:

- The number of different spikes after ignoring duplicate is 4.
- The included angle between the two diagonal circles' connecting lines is within a specific pixel range. *k* is the slope of a line and α is the angle.
  (8) $$\begin{array}{l} \begin{array}{c} tan\left(\alpha \right)=|\left(k_{2}-k1\right)/\left(1+k_{1}\cdot k_{2}\right)|\\ s.t.\;tan\left(\alpha_{1}\right)<tan\left(\alpha \right)<tan\left(\alpha_{2}\right) \end{array} \end{array}$$
- The length difference between two diagonal circles' connecting lines is within a specific pixel range, where *l* means the length.
  (9) $$\begin{array}{l} \begin{array}{l} l_{1}<|\sqrt{{\left(x_{1}-x_{4}\right)}^{2}+{\left(y_{1}-y_{4}\right)}^{2}}\\ \;-\sqrt{{\left(x_{2}-x_{3}\right)}^{2}+{\left(y_{2}-y_{3}\right)}^{2}}|<l_{2} \end{array} \end{array}$$
  Thus, the whole procedure of the proposed SNN for asynchronous Hough circle transform is presented as Algorithm 2.

**Algorithm 2:** Event-based multi-circle detecting asynchronously in the SNN.

Utilize the spatiotemporal filter in section 3 after raw events				
Initialize the timestamps *t*_*i*_ and parameters of SNN				
**for** every event *e*_*i*_ = (*t*_*i*_, *x*_*i*_, *y*_*i*_, *s*_*i*_) in the events queue **do**				
	**for** every radius *r* in the range of object size (from *r*_*min*_ to *r*_*max*_) **do**			
		**for** every degree θ_*i*_ in the range of 360 **do**		
			Calculate X-coordinate of the center	
*x*_*c*_ = *argmin*|*x*_*c*_ − (*x*_*i*_ + *r* · *cosθ*_*i*_)|				
			Calculate Y-coordinate of the center *y*_*c*_ = *argmin*|*y*_*c*_ − (*y*_*i*_ + *r* · *sinθ*_*i*_)|	
			**if** |*x*_*c*_| < *columns* and |*y*_*c*_| < *rows* **then**	
				Input the spike *s*_*i*_ to the neuron (*y*_*c*_, *x*_*c*_) at
*t*_*i*_ and upgrade it with Algorithm 1				
	**if** *t*_*i*_ − *t*_*n*−*k*+1_ ≥ *k* **then**			
		Generate all the output spikes (*y*_*c*_, *x*_*c*_) between the		
short period *t*_*n*−*k*+1_*t*_*n*+1_				
		**if** spikes meet the rule of features above **then**		
			Output the 4 points	
(*y*_*c*1_, *x*_*c*1_), (*y*_*c*2_, *x*_*c*2_), (*y*_*c*3_, *x*_*c*3_), (*y*_*c*4_, *x*_*c*4_) to an array				
			Calculate the pose of DVS in world with the	
following P4P algorithm				
		Reset SNN to 0 and renew the matrix of timestamps		
*t*_*n*−*k*+1_ ← *t*_*i*_				

### 4.2. Perspective-4-Point Algorithm for Pose Estimation

PnP algorithm is a method to estimate the pose of the camera relative to the world coordinate system by knowing the 3D coordinates of n points in space, the corresponding 2D point coordinates, and the internal parameter matrix of the camera. Considering the feasibility, four LED light sources are used to constitute the perspective-4-point (P4P) problem (Horaud et al., 1989). The problem is cast into solving an unknown biquadratic polynomial equation. It was developed as part of a monocular object recognition system (Horaud, 1987).

In the condition that 3D coordinates of 4 points (*P*1, *P*2, *P*3, *P*4) in the world coordinate system were known, the 2D coordinates of 4 points(*p*1, *p*2, *p*3, *p*4) in pixel coordinate system were calculated by Algorithm 2 and the internal parameter matrix *K* of the camera was calibrated, the camera pose relative to the world coordinate system can be calculated as follow.


(10) $$\begin{array}{l} \left[\begin{array}{c} X_{c}\\ Y_{c}\\ Z_{c} \end{array}\right]=\left[\begin{array}{c} R\;t \end{array}\right]\left[\begin{array}{c} X_{w}\\ Y_{w}\\ Z_{w} \end{array}\right] \end{array}$$

In the formula, (*X*_*c*_, *Y*_*c*_, *Z*_*c*_) and (*X*_*w*_, *Y*_*w*_, *Z*_*w*_) represent both the camera and world coordinate system. Besides, *R* is the rotation matrix and *t* is the translation vector, which describe the transformation relationship between the two coordinate systems.

In order to solve the P4P problem faster on-chip, the algorithm of Gao et al. (2003) combined with the projection method was used. Firstly, four groups of solutions are calculated with three points to obtain four rotation matrices and translation matrices. Then the result [*R t*] can be calculated according to the formula:


(11) $$\begin{array}{l} \left(\begin{array}{c} u\\ v\\ 1 \end{array}\right)\sim \left[\begin{array}{ccc} f_{x} & 0 & c_{x}\\ 0 & f_{y} & c_{y}\\ 0 & 0 & 1 \end{array}\right]\cdot \left[\begin{array}{cccc} r_{11} & r_{12} & r_{13} & t_{1}\\ r_{21} & r_{22} & r_{23} & t_{2}\\ r_{31} & r_{32} & r_{33} & t_{3} \end{array}\right]\cdot \left[\begin{array}{c} X\\ Y\\ Z\\ 1 \end{array}\right] \end{array}$$

Where *f*_*x*_, *f*_*y*_, *c*_*x*_, *c*_*y*_ are parameters of the DVS, *u, v* are coordinates in image, *R* is the rotation matrix, *t* is the translation vector and (*X, Y, Z*) is the world coordinates of the 4th points.

After substituting (*X, Y, Z*) into the formula, four projections (*u, v*) in the image are obtained. The matrix [*R t*]with the slightest projection error is the right solution. Therefore, the pose is obtained as well as the relative depth between AUV and docking ring, which guides the AUV during docking task.

## 5. Experiments and Results

The proposed multiple circle detection method was evaluated in both the simulator and real scenario, while the effectiveness of estimating the position of AUV was evaluated.

**Figure 4:**
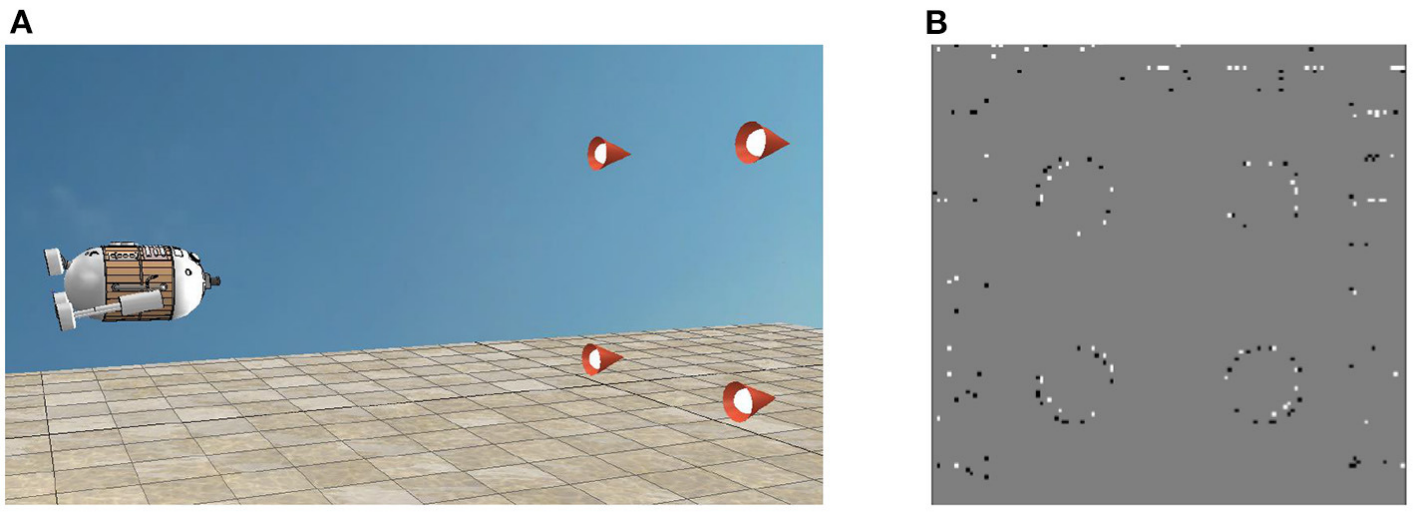
**(A)** AUV and targets in V-REP. **(B)** Events of targets in V-REP.

In the simulation, the docking of AUV is carried out in V-REP to verify the practicability of the proposed positioning algorithm in SNN. The scene and the events are displayed respectively as Figures 4A,B. In general, the visual guidance system consists of four parts: 4 circular LED lights constrained by rectangle, a dynamic vision sensor, a computer and AUV. To achieve docking, AUV's three degrees-of-freedom (DOF) in transverse *X*, longitudinal *Y*, and vertical *Z* direction could be artificially controlled. Besides, the DVS was fixed on the head of the AUV, with the visual field being limited to 65 degrees. In addition, the targets remained stationary in the scene while AUV approached them vertically according to the relative position deviation. Moreover, the computer detected targets and estimated AUV's position by using SNN and P4P algorithms. In this case, X-axis and Y-axis were parallel to the target plane, while Z-axis was perpendicular. Note that Z-axis is less than 0 according to the right-handed coordinate system. The initial position (*X, Y, Z*) in millimeters of the four targets are (1000, 2000, 0), (1000, 4000, 0), (3000, 2000, 0), (3000, 4000, 0), and AUV's initial placement point is (2000, 3000, −3000). In order to compare the estimation difference among 3 DOF, AUV's position in the X direction is limited to 2000.

**Figure 5:**
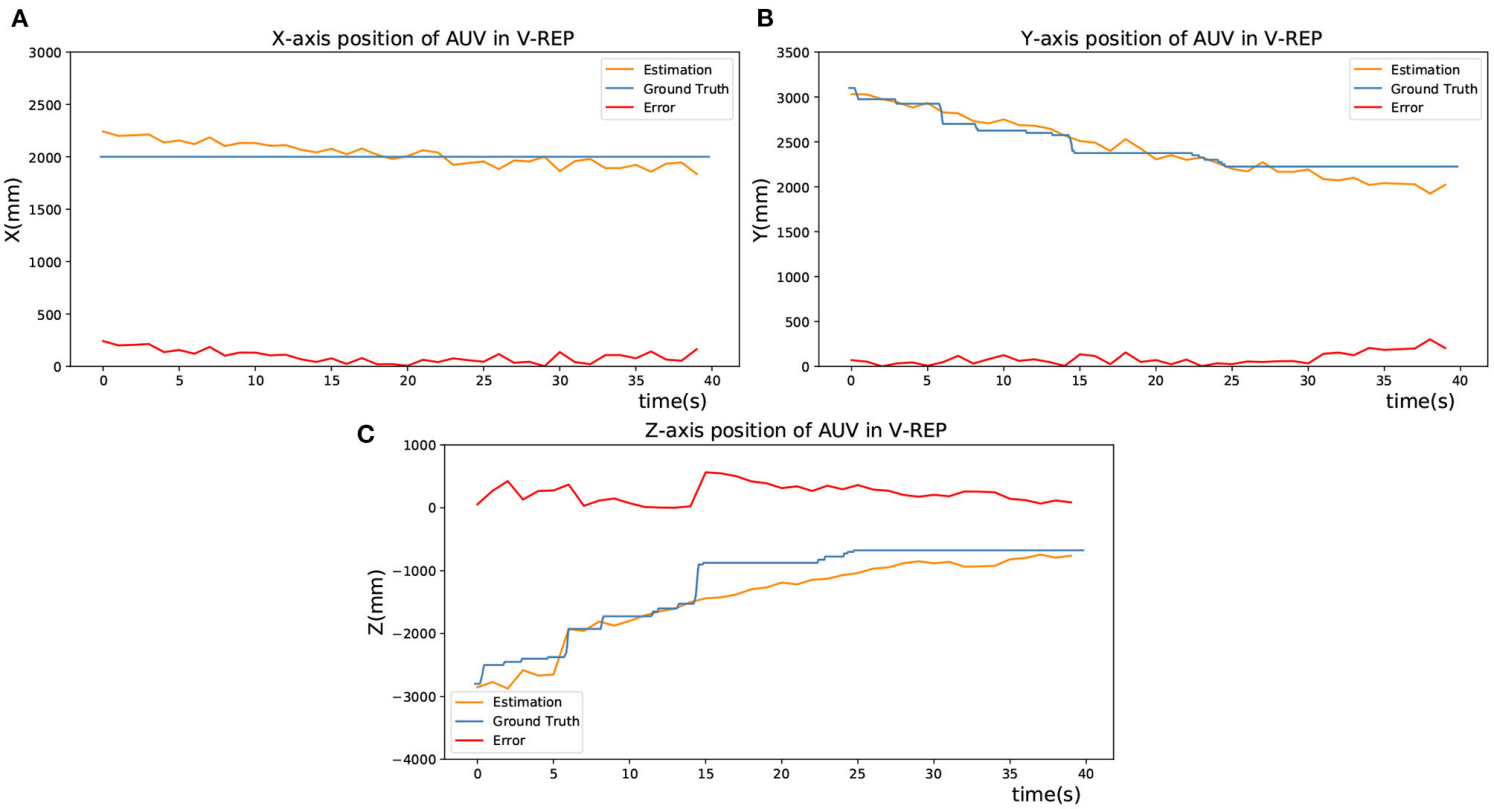
Estimation of the AUV's position in V-REP. **(A)** X-axis position of AUV in V-REP. **(B)** Y-axis position of AUV in V-REP. **(C)** Z-axis position of AUV in V-REP.

During the docking process, the motion command was sent to AUV to approach the targets gradually. When AUV was approaching, the position deviation of its Z-direction would gradually increase up to 0, which means the relative depth is getting closer. In Figure 5, the blue lines represent the actual spatial trajectory of the AUV relative to targets in 3 DOF, which is recorded by a graph fixed on AUV. The yellow lines represent the estimated position of AUV by using the SNN and P4P algorithm. Meanwhile, red lines represent the deviation of estimation during the AUV's movement, which is obtained by comparing the difference between the ground truth and estimation. As a result, Figure 5 verifies the feasibility of the visual positioning method.

In the pool experiments, the detection effects of the frame-based Gradient Hough Transform (GHT) (Chen et al., 2012) and the proposed SNN based method were compared.

**Figure 6:**
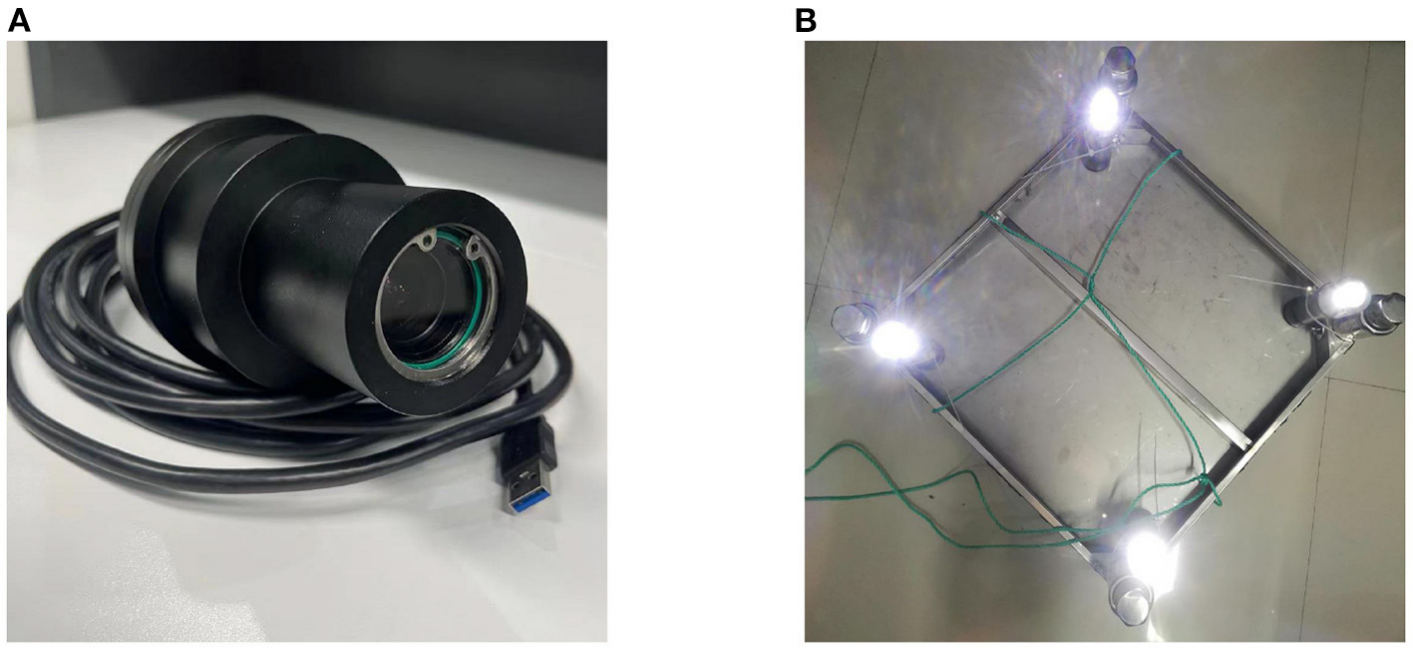
The experimental equipments. **(A)** DVS with waterproof shell. **(B)** Targets with lights and steel plate.

The visual guidance system in the experimental pool consists of three parts: 4 LED light sources, a dynamic vision sensor and a computer. In the pool, X-axis and Y-axis were parallel to the target plane, while Z-axis was perpendicular. Four underwater circular lights were bound vertically to the corners of a rectangular plate and kept stationary underwater. In addition, the DVS used in experiment is produced by IniVation, which has a 320 × 240 spatial resolution, 1μ*s* temporal accuracy and the internal parameters matrix (257.3, 164.1, 255.4, 130.4). Meanwhile, a waterproof shell coated with a metal oxide layer is processed. The experimental equipment is displayed as Figure 6. And to simulate the docking motion of AUV, the DVS was made close to or away from the stationary targets. During the process, the computer parsed DVS's data, detected targets and estimated DVS's position by using SNN and P4P algorithms.

**Figure 7:**
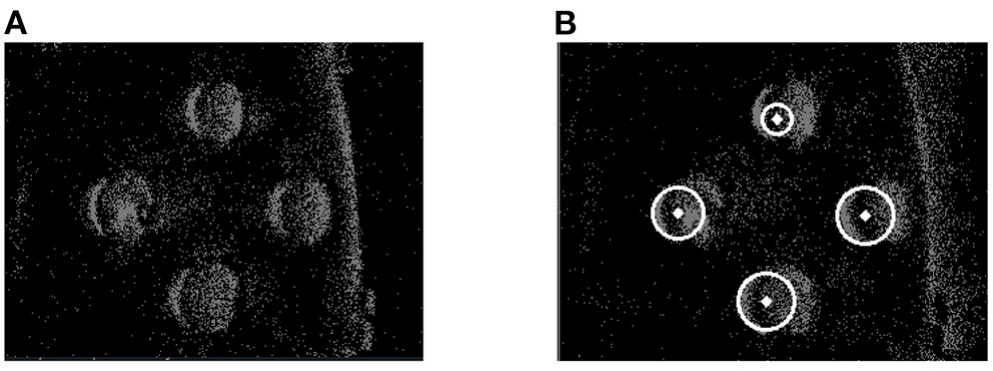
Results of frame-based GHT. **(A)** Raw event frame. **(B)** Effect of detection.

In order to verify the detection effect of the SNN algorithm, it was compared with a baseline method. The frame-based Gradient Hough Transform(GHT) is a classical method for detecting circles and utilized as baseline. During the experiment for GHT, raw event stream in a period was first accumulated as a pseudo-frame. Then it was changed to a grayscale image, filtered by the median, operated by morphological open, and extracted the edges. Later the OpenCV library was utilized to realize the GHT method, and the parameters of GHT were set as Table 1. Detection effects of GHT were as Figure 7.

**Table 1:** The parameters of pseudo-frame gradient Hough circle transform.

**Parameter**	**Value**	**Unit**
Rows	240	pixels
Columns	320	pixels
Time of one frame	50,000	μs
Minimum distance between objects	60	pixels
High threshold of edge detection	40	
Accumulator threshold	12	
Range of radius	5–26	pixels

**Figure 8:**
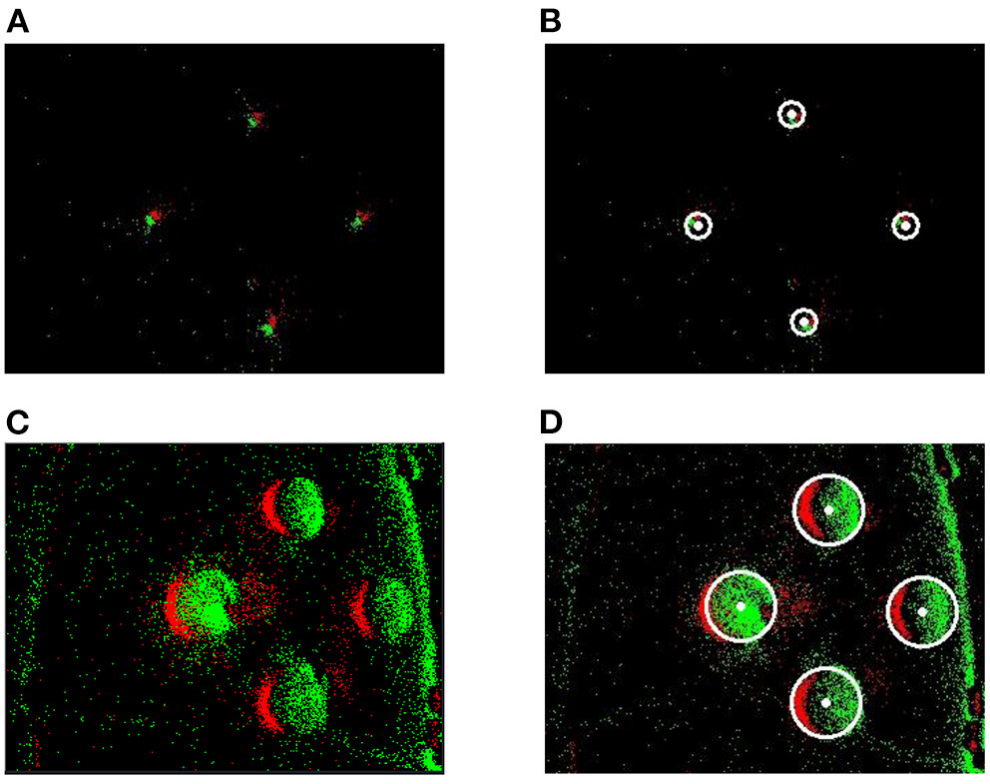
The detection effect of SNN. **(A)** Raw events of far targets. **(B)** Detection of far targets. **(C)** Raw events of near targets. **(D)** Detection of near targets.

In the proposed method, the event stream was first put into the spatiotemporal filter compared to GHT. The time interval (*dt*) regarding the spatiotemporal neighbors and the number (*n*) of supporting pixels were configured as 1 ms and 76,800, respectively. A 240 × 320 SNN was established according to the DVS's resolution. Then the proposed Asynchronous Hough Circle Transform in SNN (section 4.1) was implemented in Python. The parameters in Table 2 were set to SNN, and the detected objects were exhibited in Figure 8 with the camera approaching targets. In Figure 8, the green points represent positive events which generate positive spikes to SNN, while the red points represent negative events.

**Table 2:** The parameters of SNN based asynchronous Hough transform.

**Parameter**	**Value**	**Unit**
Rows	240	pixels
Columns	320	pixels
Spike threshold *v*_*th*_	150	mVolts
Rate of decay λ	0.0006	mVolts/μ s
Margin of lateral inhibition *m*	60	pixels
Refractory period *t*_*i*_ − *t*_*i*−1_	1	μs
Range of radius *r*	5–26	pixels
Time interval for counting spikes *k*	50,000	μs
Spike value *s*_*i*_	1	mVolts

Table 3 reports the statistics of detection results by using SNN. With time increasing, massive input events from the receptor layer generated fewer output spikes to the output layer, which indicated the changes of targets in the image. Therefore, the generated time, end time and the number of spikes were recorded for quantitative analysis. As we can see, the positions (*X, Y*) of all targets can be accurately detected and tracked.

**Table 3:** Quantitative analysis of multiple circle detection by SNN.

**Time**	**Input**	**Output**	**Spike time**	**X position**	**Y position**
**(ms)**	**events**	**spikes**	**(event order)**	**of 4 targets**	**of 4 targets**
**Start/end**	**numbers**	**numbers**	**Start/end**	**(pixel)**	**(pixel)**
0 50	11024	2992	81024 85480	150 203 204 273	116 184 46 123
50 100	11432	2008	92240 97896	139 202 207 274	117 178 55 121
100 150	11792	2480	103440 108784	146 210 216 296	110 187 46 159
150 200	11760	2640	115192 121144	152 216 217 283	121 188 46 118
200 250	11520	2144	126952 132696	154 218 221 285	130 181 50 118

**Figure 9:**
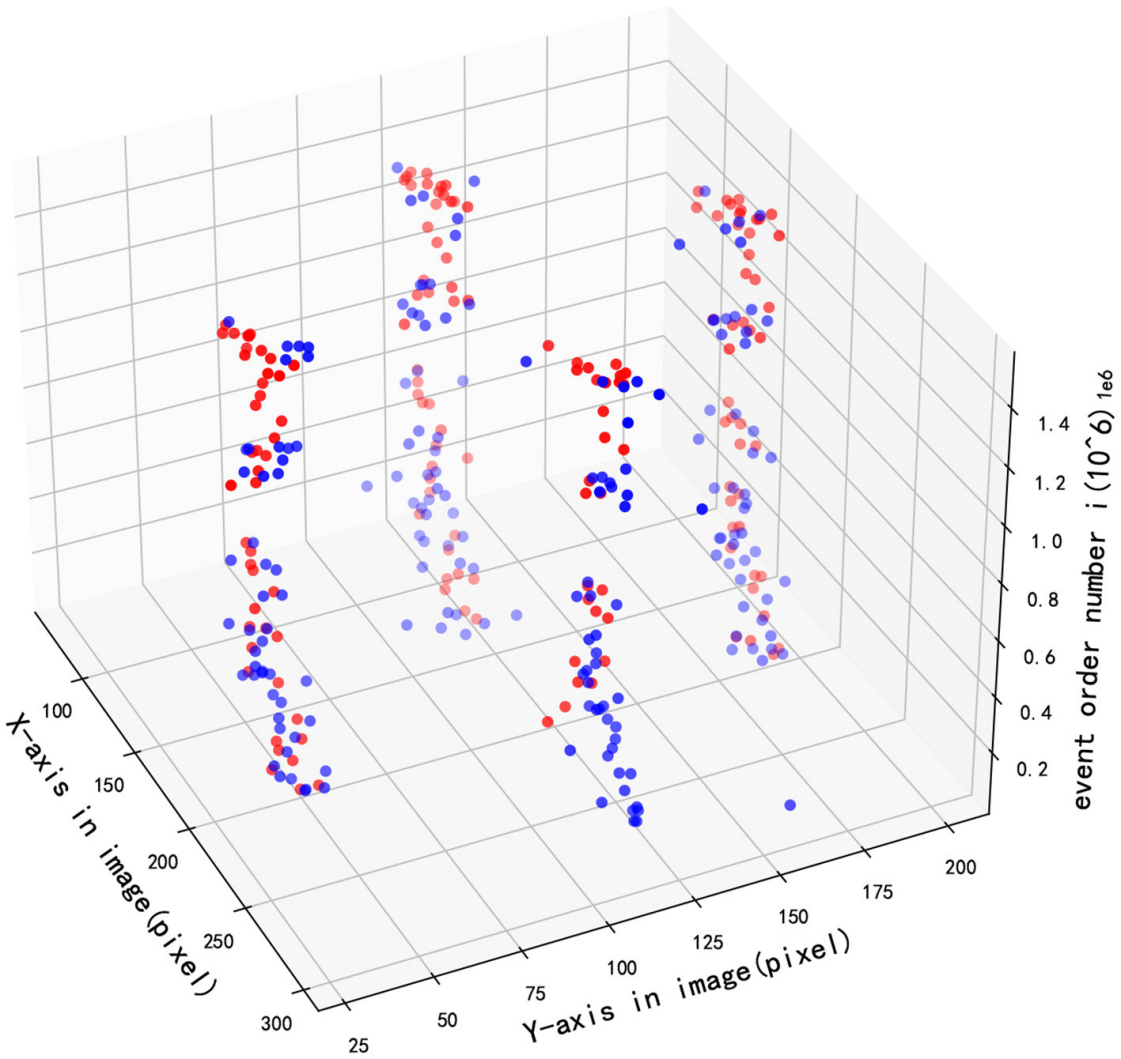
Multi-target tracking effect in image.

Figure 9 demonstrates the detection performance between two methods when the pixel position of multiple targets changes with event sequence, where red and blue points respectively present the results of GHT and SNN. As illustrated in the figure, the accuracy of multi-target tracking of the two algorithms is close.

**Figure 10:**
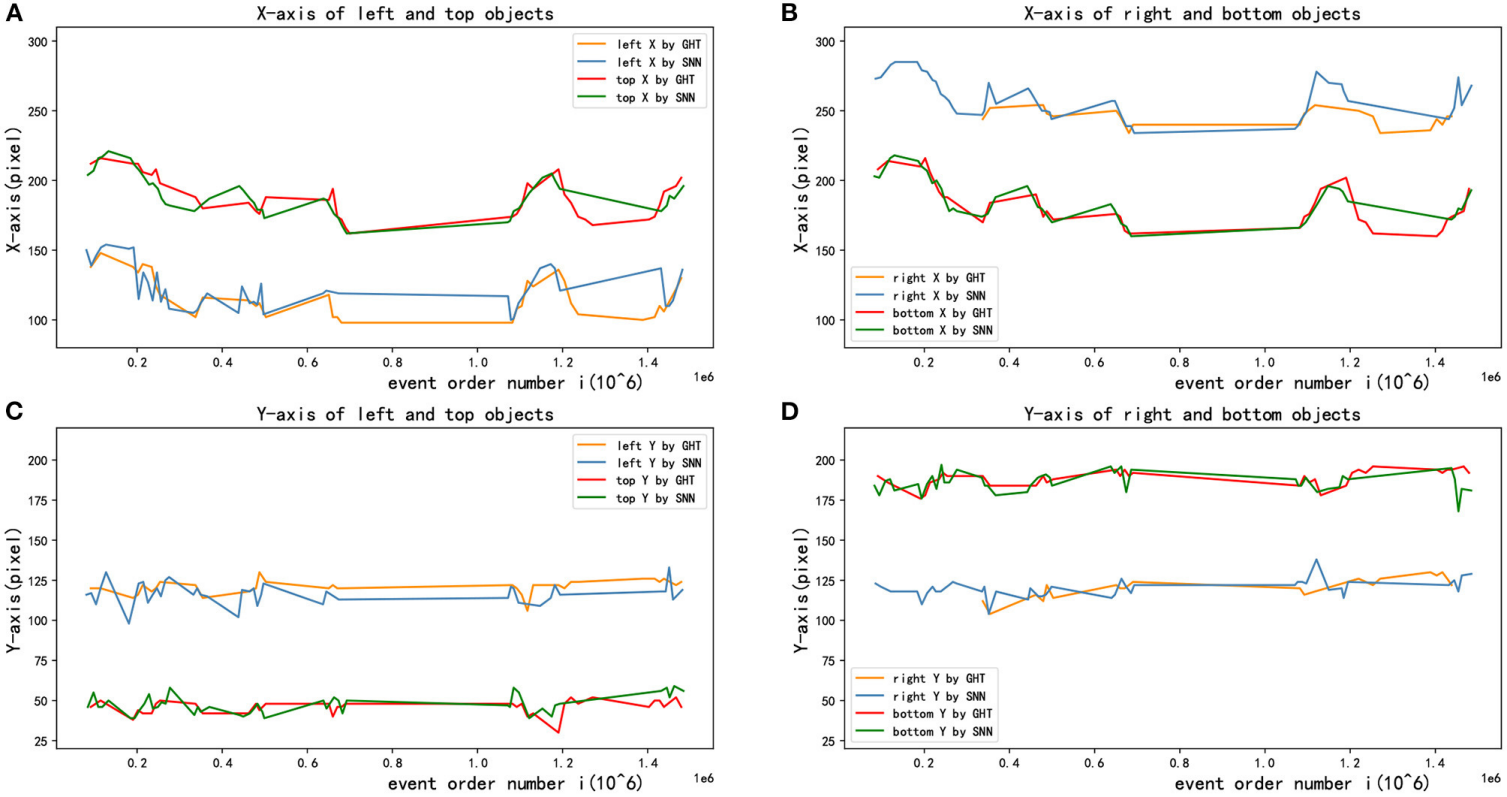
Multi-target tracking effects in X and Y direction. **(A)** X-axis of left and top objects. **(B)** X-axis of right and bottom objects. **(C)** Y-axis of left and top objects. **(D)** Y-axis of right and bottom objects.

**Figure 11:**
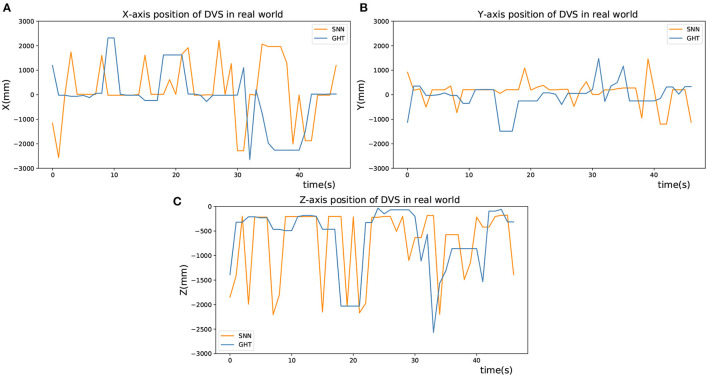
Position estimation in real world. **(A)** X-axis position of DVS in real world. **(B)** Y-axis position of DVS in real world. **(C)** Z-axis position of DVS in real world.

As presented in Figure 10A, the X-coordinate trajectories of the upper and the lower circle are basically the same, and so on in Y-coordinate of the left and right circle, which shows the accuracy of target detection. Besides, Figure 10B shows the detected X-axis trajectories of right and bottom objects by two different methods, Figure 10C shows the Y-axis trajectories of left and top objects, and Figure 10D shows the Y-axis trajectories of right and bottom objects. In addition, the detection results of the GHT and SNN methods are almost equal. However, the jitter of the curve of SNN method is smaller than GHT method. After obtaining image coordinates at the detection stage, relative position in the real scenario was estimated by the P4P algorithm. The estimation performance from two methods is displayed in Figure 11, where X and Y axes are positions parallel to the target plane, and Z-axis represents relative depth. Note that Z-axis position is always less than 0 because the camera only appears on one side of the target plane. It can be seen from the figure that two methods have similar results for position estimation.

## 6. Conclusion

In this paper, a multiple circles detection and relative pose estimation method was proposed, which combined monocular DVS and SNN for AUV docking. The proposed method not only avoids motion blur caused by frame-based recognition but also adapts to high exposure at close range and reduces data redundancy effectively, which utilizes the biological characteristics of SNN and hardware features of DVS. We focus on calculating asynchronous Hough mappings and constructing an SNN model. The accuracy of our method is compared with a frame-based method. Pool experiments and simulations are carried out to verify the effectiveness of the method. In future work, stereo event-based cameras are considered to combine with SNN to improve the recognition accuracy and speed.

## Data Availability Statement

The original contributions presented in the study are included in the article/supplementary material, further inquiries can be directed to the corresponding author/s.

## Author Contributions

FZ and YZ present the idea in this article and write the paper together. LC and ZW provide the technical support and help to complete the experimental verification. All authors contributed to the article and approved the submitted version.

## Funding

This study was supported by the National Natural Science Foundation of China (52171322), the National Key Research and Development Program (2020YFB1313200), and the Fundamental Research Funds for the Central Universities (D5000210944).

## Conflict of Interest

The authors declare that the research was conducted in the absence of any commercial or financial relationships that could be construed as a potential conflict of interest.

## Publisher's Note

All claims expressed in this article are solely those of the authors and do not necessarily represent those of their affiliated organizations, or those of the publisher, the editors and the reviewers. Any product that may be evaluated in this article, or claim that may be made by its manufacturer, is not guaranteed or endorsed by the publisher.
